# Corticosteroid Use in the Treatment of COVID-19: A Multicenter Retrospective Study in Hunan, China

**DOI:** 10.3389/fphar.2020.01198

**Published:** 2020-08-12

**Authors:** Yiming Ma, Huihui Zeng, Zijie Zhan, Huanhuan Lu, Zihang Zeng, Chenjie He, Xiangming Liu, Chen Chen, Qingwu Qin, Jia He, Zhiguo Zhou, Peng Huang, Mingyan Jiang, Dingding Deng, Xin Liao, Zhi Xiang, Xiaoying Huang, Yan Chen, Ping Chen

**Affiliations:** ^1^Department of Respiratory and Critical Care Medicine, The Second Xiangya Hospital, Central South University, Changsha, China; ^2^Department of Thoracic Surgery, The Second Xiangya Hospital, Central South University, Changsha, China; ^3^Department of Cardiology, The Second Xiangya Hospital, Central South University, Changsha, China; ^4^Department of Respiratory Medicine, The First Hospital of Changsha, Changsha, China; ^5^Department of Respiratory Medicine, Zhuzhou Central Hospital, Zhuzhou, China; ^6^Department of Respiratory and Critical Medicine, Xiangtan Central Hospital, Xiangtan, China; ^7^Department of Respiratory Medicine, The First Affiliated Hospital of Shaoyang University, Shaoyang, China; ^8^Department of Respiratory Medicine, The Central Hospital of Shaoyang, Shaoyang, China; ^9^Department of Respiratory Medicine, Huaihua First People’s Hospital, Huaihua, China; ^10^Department of Respiratory Medicine, Loudi Central Hospital, Loudi, China

**Keywords:** COVID-19, corticosteroid, treatment, antibiotic, viral shedding

## Abstract

**Background:**

Coronavirus disease 2019 (COVID-19) has developed into a worldwide pandemic. This study aimed to retrospectively describe the use of corticosteroids in treating COVID-19.

**Methods:**

For this multicenter retrospective study, medical records from 488 symptomatic COVID-19 patients were reviewed. Patients were divided into severe and nonsevere groups. Baseline characteristics, signs and symptoms, laboratory findings, treatments, and disease outcomes were compared. Specific data for corticosteroid treatment were further analyzed.

**Results:**

Four hundred fifty COVID-19 patients were included in this study, including 82 severe patients and 368 nonsevere cases. Out of the 450 patients, 126 (28.0%) received corticosteroid treatment. In the 126 patients treated with corticosteroids, the median daily dose of corticosteroid therapy was 56.6 [interquartile range (IQR): 40.0–78.4] mg and median corticosteroid therapy duration was 5.0 (IQR: 3.0–7.0) days. Among nonsevere cases, patients treated with corticosteroids were significantly older in comparison with patients who did not receive corticosteroid treatment (*p*<0.01); the proportion of patients receiving antibiotic therapy in the corticosteroid group was significantly higher than that in the noncorticosteroid group (*p*<0.001); hospitalization length and duration of viral shedding were significantly longer in patients in the corticosteroid group than in the noncorticosteroid group after adjusting for age, sex, and comorbidities (*p*<0.05). In severe cases, patients treated with corticosteroids were significantly older and comorbidities at admission were significantly more common in patients receiving corticosteroid treatment (*p*<0.05); the proportion of patients receiving antibiotic therapy in the corticosteroid group was significantly higher than that in the noncorticosteroid group (*p*<0.001); no significant difference in hospitalization length or viral shedding duration was found between two groups after adjusting for age, sex, and comorbidities (*p*>0.05).

**Conclusion:**

Our study indicates that corticosteroids are regarded as one of treatments in the general clinical practice of COVID-19. However, corticosteroid use may be accompanied by increased use of antibiotics, longer hospitalization, and prolonged viral shedding.

## Introduction

In December 2019, a series of novel coronavirus-related pneumonia cases were reported in Wuhan, China. The disease was named as coronavirus disease 2019 (COVID-19) by World Health Organization (WHO) (WHO). A report of 72,314 cases from the Chinese Center for Disease Control and Prevention (CDC) demonstrated that severe or critical COVID-19 cases accounted for 19% of all cases and the overall case-fatality rate was 2.3% (Wu and McGoogan, 2020), which has posed serious threats to both human health and public health services. The COVID-19 epidemic has spread rapidly, developing into a worldwide pandemic (Bedford et al., 2020). Consequently, it is crucial to establish a reasonable and effective treatment strategy.

Corticosteroids, as widely used antiinflammatory agents and immunosuppressants, play an important role in the treatment of critical illness. Previous studies demonstrated that corticosteroid was used in treating coronavirus diseases, including severe acute respiratory syndrome (SARS) (Jang et al., 2004; Chen et al., 2006; Yam et al., 2007) and Middle East respiratory syndrome (MERS) (Arabi et al., 2018; Alfaraj et al., 2019); however, the safety and effectiveness remained controversial. Studies have shown that cytokine storm caused by the massive release of cytokines was the main cause of death in some cases of SARS (Huang et al., 2005) and MERS (Kim et al., 2016; Min et al., 2016); and corticosteroids may have a certain antagonistic effect on cytokine storm (Jung et al., 2007; Zhang et al., 2008). Nevertheless, the use of corticosteroids can also lead to prolonged coronavirus clearance (Lee et al., 2004) and adverse outcomes (Auyeung et al., 2005).

On Jan 28, 2020, WHO released interim guidance for the clinical management of COVID-19 and did not list corticosteroids as a recommended treatment (World Health Organization, 2020). On Feb 8, 2020, an expert consensus from China was published and provided specific guidance for the use of corticosteroids in the treatment of COVID-19, including indications, usage, dosage, and course (Zhao et al., 2020). However, the evidence for corticosteroid treatment in general clinical practice when treating COVID-19 was insufficient. Thus, this study aimed to retrospectively describe the use of corticosteroids in treating COVID-19 and thereby provided reference materials for clinicians.

## Methods

### Study Design and Participants

For this multicenter retrospective study in Hunan, China, the medical records of 488 symptomatic COVID-19 patients who were admitted to the Public Health Treatment Center of Changsha, The Central Hospital of Shaoyang, People’s Hospital of Lucheng District, Huaihua First People’s Hospital, Xiangtan Central Hospital, and Loudi Central Hospital between January 23^rd^, 2020 and March 8^th^, 2020 were reviewed. Subjects fulfilling eligible criteria were enrolled. The inclusion criteria included: (1) laboratory-confirmed COVID-19 cases based on the results of next-generation sequencing or real-time reverse transcription polymerase chain reaction (RT-PCR) methods, as described previously (Huang et al., 2020; 2) symptomatic for COVID-19 (fever, cough, expectoration, shortness of breath, rhinorrhea, sore throat, myalgia, chill, fatigue, headache, chest pain, conjunctival congestion, nasal congestion, nausea, vomiting, and diarrhea) (Chen et al., 2020; Guan et al., 2020; Huang et al., 2020), and fever was defined as an axillary temperature ≥37.3°C. Exclusion criteria were: (1) aged less than 18 years and (2) missing treatment details during hospitalization. All patients were divided into severe and nonsevere groups. Severe COVID-19 case was defined as dyspnea, respiratory frequency ≥30/min, blood oxygen saturation ≤93%, PaO2/FiO2 ratio <300, and/or lung infiltrates >50% of the lung field within 24–48 h; critical COVID-19 case was defined as patients with respiratory failure, septic shock, and/or multiple organ dysfunction/failure (National Health Commission of the People’s Republic of China). And patients meeting the definition of severe COVID-19 case or critical COVID-19 case were categorized into the severe group, otherwise, patients were included in the nonsevere group. All sites followed the unified treatment guidelines for COVID-19 from National Health Commission of China, which have been updated since the start of the pandemic. The most updated Chinese management guideline (7^th^ edition) suggests that for patients with progressive deterioration of the oxygenation index, rapid progression on imaging, and hyperactive inflammatory reaction, corticosteroids should be used for a short period of time (3–5 days) as appropriate, and the dose should not exceed 1–2 mg/kg/day of methylprednisolone (National Health Commission of the People’s Republic of China). The study protocol was approved by the institutional ethics committee of The Second Xiangya Hospital (2020-010) and got approvals from all other participant sites. The informed consent was waived because of the retrospective design.

### Procedures and Measurements

Baseline characteristics (age, sex, and comorbidities), signs and symptoms at admission (fever, cough, expectoration, dyspnea, temperature, and respiratory rate), laboratory findings at admission (arterial blood pH, arterial blood PaO2, white blood cell count, neutrophils, lymphocyte count, and serum lactate dehydrogenase), treatments during admission (corticosteroids, antiviral agents, antibiotics, antifungal agents, noninvasive mechanical ventilation, invasive mechanical ventilation, extracorporeal membrane oxygenation, and continuous renal replacement therapy) and disease outcomes (length of hospitalization, length of virus shedding and death) of all eligible COVID-19 patients were obtained. A unified standardized form was used to retrieve relevant data and all data were collected by a group of trained respiratory doctors. All data were checked by two physicians. Regarding corticosteroid treatment, specific data (number of patients receiving corticosteroid treatment, time from illness onset to corticosteroid therapy, time from admission to corticosteroid therapy, accumulative dose of corticosteroid therapy, mean daily dose of corticosteroid therapy, duration of corticosteroid therapy, types of corticosteroids, and routes of corticosteroid administration) were further analyzed. Doses of corticosteroid were uniformly converted to methylprednisolone by considering 4 mg of methylprednisolone and 5 mg of prednisolone as equivalent doses (Chen et al., 2006). The illness onset time was defined as the day of COVID-19 related symptoms appearing for the first time. The duration of viral shedding was defined as the time from the date of symptom onset to the date when two consecutive throat-swab with an interval more than 24 h were negative for viral species and there was no subsequent positive test (Zuo et al., 2020).

### Statistical Analysis

Continuous variables with a normal distribution are expressed as the mean and standard deviation (SD), while the median and interquartile range (IQR) are used to describe continuous variables with a nonnormal distribution. Categorical variables are presented as frequencies and percentages. The Chi-square test or Fisher exact test were employed to compare categorical variables. The independent sample T test was used to compare continuous variables with a normal distribution, and the Mann-Whitney U test was conducted to compare continuous variables with a nonnormal distribution. In order to adjust for confounding factors (age, sex, and comorbidities), we adopted propensity score 1:1 matching method to match patients receiving corticosteroid treatment with patients not receiving corticosteroid treatment within a caliper of 0.2 standard deviation of logit of propensity score in severe group and nonsevere group (patient numbers and characteristics of the cohorts before and after matching are available in **Supplemental Table 1**); then Mann-Whitney U test was used when comparing disease outcomes (length of hospitalization, length of viral shedding) between corticosteroid group and noncorticosteroid group. SPSS Windows Version 23.0 (IBM Corporation, Armonk, NY, USA), R software version 3.6.2 (R Foundation for Statistical Computing), and GraphPad Prism Version 7.04 (GraphPad, San Diego, CA) were used to perform statistical analysis. A two-side P-value of less than 0.05 was defined as statistically significant.

## Results

**Figure 1:**
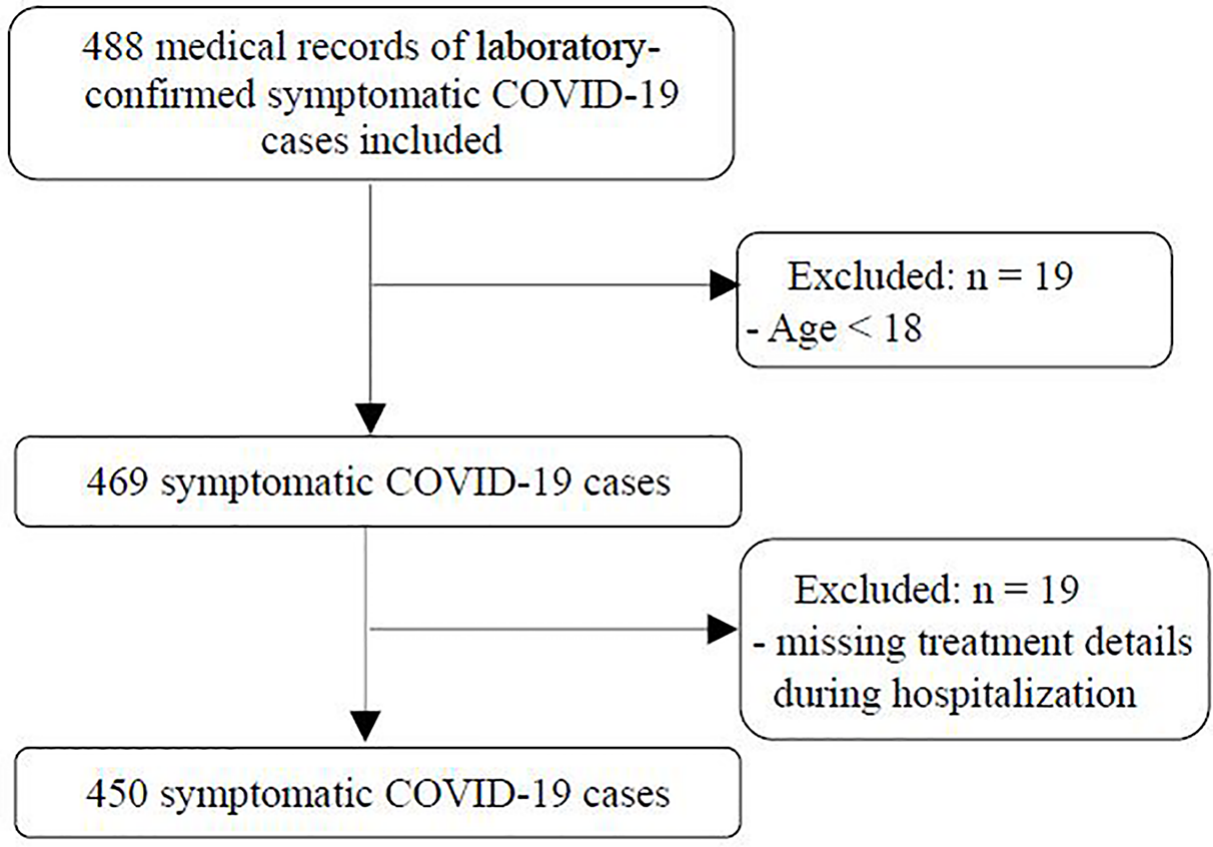
Flow chart of this study.

A total of 450 symptomatic COVID-19 patients from six centers who fulfilled the eligible criteria were included in this retrospective study. A flow chart of this study is shown in **Figure 1**. Among the 450 patients, the mean age was 46.2 ± 15.1 years, 228 (50.7%) were males, 128 (28.4%) had one or more comorbidities (hypertension, diabetes mellitus, cardiovascular disease, chronic liver disease, chronic kidney disease, cerebrovascular disease, chronic obstructive pulmonary disease, malignancy and rheumatic disease), and there were 82 severe cases and 368 nonsevere cases. The general characteristics of severe cases and nonsevere cases are shown in **Table 1**.

**Table 1:** General characteristics of severe and nonsevere coronavirus disease 2019 (COVID-19) patients.

Variables	Overall (N=450)	Severe group (N=82)	Nonsevere group (N=368)	*P*-value
Baseline characteristics				
Age (years)	46.2 ± 15.1	56.9 ± 15.5	43.9 ± 14.0	<0.001
Sex, male	228 (50.7)	48 (58.5)	180 (48.9)	0.115
Any comorbidity	128 (28.4)	49 (59.8)	79 (21.5)	<0.001
Hypertension	75 (16.7)	31 (37.8)	44 (12.0)	<0.001
Diabetes mellitus	45 (10.0)	18 (22.0)	27 (7.3)	<0.001
Cardiovascular disease	22 (4.9)	10 (12.2)	12 (3.3)	0.002
Chronic liver disease	11 (2.4)	3 (3.7)	8 (2.2)	0.695
Chronic kidney disease	1 (0.2)	1 (1.2)	0 (0)	0.182
Cerebrovascular disease	11 (2.4)	5 (6.1)	6 (1.6)	0.048
Chronic obstructive pulmonary disease	10 (2.2)	6 (7.3)	4 (1.1)	0.002
Malignancy	5 (1.1)	2 (2.4)	3 (0.8)	0.226
Rheumatic disease	2 (0.4)	1 (1.2)	1 (0.3)	0.332
Signs and symptoms at admission				
Fever	338 (75.1)	69 (84.1)	269 (73.1)	0.036
Cough	333 (74.0)	66 (80.5)	267 (72.6)	0.139
Expectoration	188 (41.8)	38 (46.3)	150 (40.8)	0.354
Dyspnea	58 (12.9)	27 (32.9)	31 (8.4)	<0.001
Temperature, ℃	36.9 (36.5–37.4)	37.2 (36.6-37.8)	36.9 (36.5–37.3)	0.004
Respiratory rate, breaths/min	20.0 (20.0–21.0)	20.0 (20.0–22.0)	20.0 (20.0–20.0)	<0.001
Laboratory findings at admission				
Arterial blood pH	7.467 (7.431–7.494)	7.480 (7.442–7.503)	7.460 (7.430–7.490)	0.030
Arterial blood PaO2, mm Hg	90.9 (76.2–115.0)	83.4 (63.3–104.3)	92.2 (78.8–115.3)	0.002
White blood cell count, *10^9^/L	4.70 (3.61–5.82)	5.08 (4.08–6.14)	4.63 (3.58–5.71)	0.064
Neutrophil count, *10^9^/L	2.93 (2.21–3.81)	3.48 (2.48–4.45)	2.84 (2.15–3.69)	0.004
Lymphocyte count, *10^9^/L	1.10 (0.80–1.54)	0.82 (0.63–1.18)	1.17 (0.86–1.60)	<0.001
Serum lactate dehydrogenase, IU/L	178.0 (146.1–220.3)	221.4 (176.2–318.8)	171.8 (142.8–209.0)	<0.001
Chest radiography at admission				
Normal	34 (7.6)	5 (6.1)	29 (7.9)	0.581
Unilateral lesion	103 (22.9)	18 (22.0)	85 (23.1)	0.823
Bilateral lesion	313 (69.6)	59 (72.0)	254 (69.0)	0.602
Corticosteroid treatment				
Number of patients receiving corticosteroid	126 (28.0)	62 (75.6)	64 (17.4)	<0.001
Time from illness onset to corticosteroid therapy, days	9.0 (6.0–11.0)	7.5 (5.0–11.0)	9.0 (7.0–11.0)	0.057
Time from admission to corticosteroid therapy, days	2.0 (1.0^–^5.0)	1.0 (1.0–3.0)	4.0 (2.0–5.8)	<0.001
Accumulative dose of corticosteroid therapy, mg	240.0 (160.0–420.0)	340.0 (230.0–680.0)	200.0 (120.0–280.0)	<0.001
Median daily dose of corticosteroid therapy, mg	56.6 (40.0–78.4)	61.5 (40.0–80.0)	40.0 (40.0–60.0)	0.002
Duration of corticosteroid therapy, days	5.0 (3.0–7.0)	6.0 (3.8–9.3)	4.0 (3.0–5.8)	0.003
Other treatments				
Antiviral therapy	447 (99.3)	82 (100.0)	365 (99.2)	1.000
Interferon	288 (64.0)	57 (69.5)	231 (62.8)	0.250
Lopinavir/Ritonavir	351 (78.0)	68 (82.9)	283 (76.9)	0.234
Arbidol	264 (58.7)	47 (57.3)	217 (59.0)	0.784
Ribavirin	24 (5.3)	6 (7.3)	18 (4.9)	0.377
Chloroquine phosphate	42 (9.3)	3 (3.7)	39 (10.6)	0.051
Antibiotic therapy	225 (50.0)	71 (86.6)	154 (41.8)	<0.001
Quinolones	190 (42.2)	53 (64.6)	137 (37.2)	<0.001
Cephalosporins	22 (4.9)	12 (14.6)	10 (2.7)	<0.001
Carbapenems	8 (1.8)	7 (8.5)	1 (0.3)	<0.001
Macrolides	4 (0.9)	1 (1.2)	3 (0.8)	0.554
Penicillins	33 (7.3)	22 (26.8)	11 (3.0)	<0.001
Linezolid	6 (1.3)	6 (7.3)	0 (0)	<0.001
Polymyxin	1 (0.2)	1 (1.2)	0 (0)	0.182
Teicoplanin	1 (0.2)	1 (1.2)	0 (0)	0.182
Antifungal therapy	5 (1.1)	4 (4.9)	1 (0.3)	0.004
Voriconazole	5 (1.1)	4 (4.9)	1 (0.3)	0.004
Noninvasive mechanical ventilation	16 (3.6)	16 (19.5)	0 (0)	<0.001
Invasive mechanical ventilation	10 (2.2)	10 (12.2)	0 (0)	<0.001
ECMO	6 (1.3)	6 (7.3)	0 (0)	<0.001
CRRT	6 (1.3)	6 (7.3)	0 (0)	<0.001
Disease outcomes				
Length of hospitalization, days	13.0 (10.0–20.0)	18.0 (13.0–24.0)	13.0 (9.0–19.0)	<0.001
Length of viral shedding, days	17.0 (13.3–24.0)	18.0 (15.0–27.5)	17.0 (13.0–23.0)	0.022
Death	3 (0.7)	3 (3.7)	0 (0)	0.006

Values are presented as median (IQR), mean ± SD or number (percentage); p values were compared by Chi-square test, Fisher’s exact test, independent sample T test, or Mann-Whitney U test.

ECMO, extracorporeal membrane oxygenation; CRRT, continuous renal replacement therapy.

### Use of Corticosteroids

**Figure 2:**
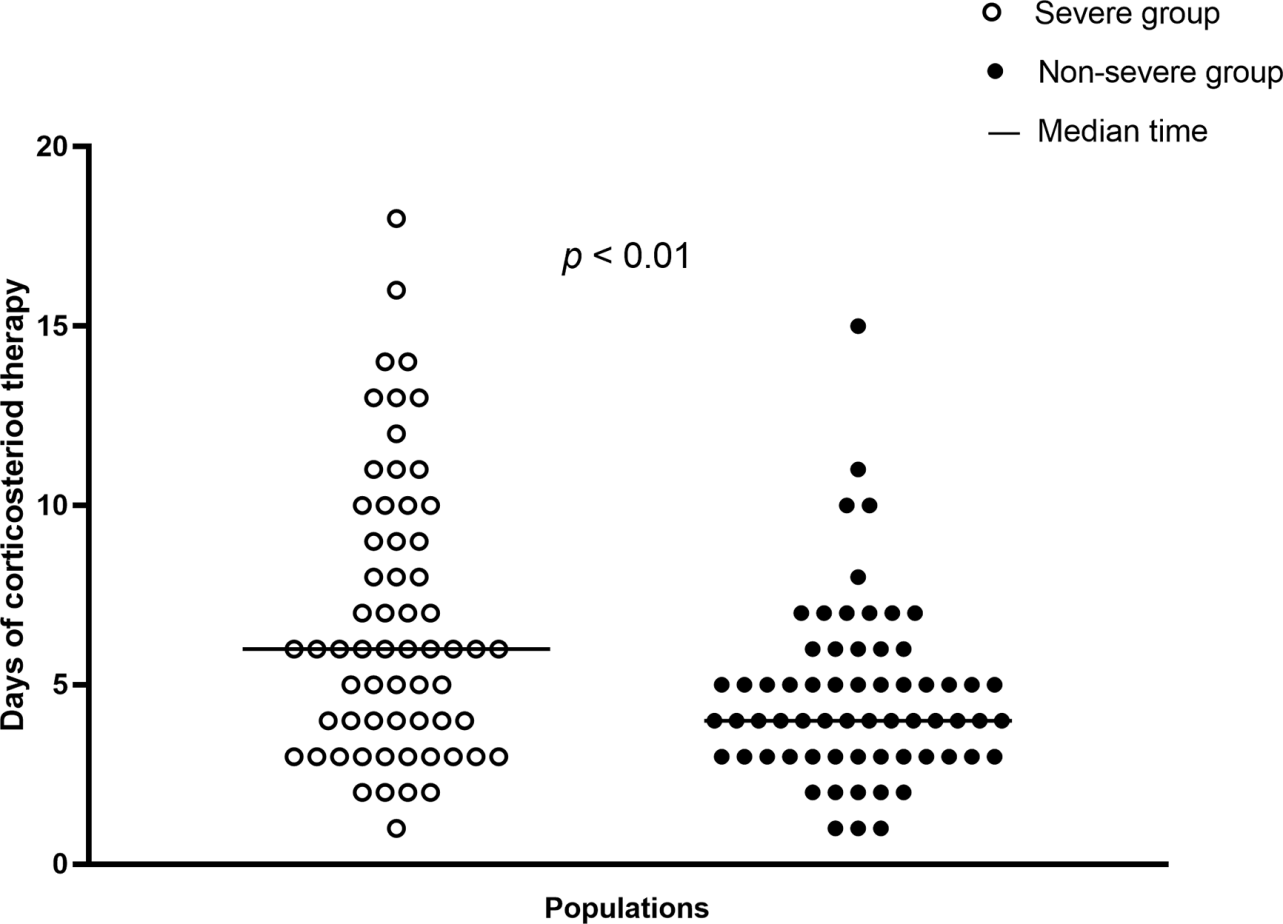
Distributions of corticosteroid treatment days in severe cases (n=62) and nonsevere cases (n=64).

In total, 126 (28.0%) patients received corticosteroid treatment. The proportion of patients in the severe group receiving such treatment was significantly higher than that in the nonsevere group (75.6% vs. 17.4%, *p*<0.001). The median time from illness onset to corticosteroid therapy in all 126 patients was 9.0 (IQR: 6.0–11.0) days. Overall, onset time that patients received corticosteroid therapy after admission was 2.0 (IQR: 1.0–5.0) days, and the corticosteroid therapy onset time after admission was significantly later in the nonsevere group (*p*<0.001). Moreover, the accumulative dose of corticosteroid therapy in the whole population was 240.0 (IQR: 160.0–420.0) mg. A significant difference in the median accumulative corticosteroid dose was found between the severe and nonsevere group (respectively, 340.0 vs. 200.0 mg, *p*<0.001). The median daily dose of corticosteroid therapy in all 126 patients was 56.6 (IQR: 40.0–78.4) mg; the severe group had a significantly higher median daily dose than the nonsevere group (61.5 vs. 40.0 mg, *p*<0.01). Regarding duration of corticosteroid therapy, the median duration in the whole population was 5.0 (IQR: 3.0–7.0) days. In contrast with the severe group, the nonsevere group demonstrated a significantly shorter median duration of therapy (4.0 vs. 6.0 days, *p*<0.01). The types of corticosteroids used included methylprednisolone and prednisolone. Overall, 125 (99.2%) patients received methylprednisolone and one (0.8%) patient received prednisolone treatment. Regarding routes of corticosteroid administration, one (0.8%) patient took corticosteroid orally, and 125 (99.2%) were given corticosteroid intravenously. The distribution of corticosteroid treatment duration in severe and nonsevere cases is demonstrated in **Figure 2**.

### Comparisons of Characteristics Between Patients Treated With and Without Corticosteroid in Nonsevere COVID-19 Cases

**Table 2** demonstrates comparisons of characteristics between patients treated with and without corticosteroid in nonsevere COVID-19 cases. Compared with patients who did not receive corticosteroid treatment, patients with nonsevere COVID-19 who received corticosteroid treatment were significantly older (*p*<0.01). Regarding signs and symptoms at admission, patients with fever were more likely to receive corticosteroid treatment (*p*<0.05). With respect to laboratory findings at admission, the arterial PaO_2_ level and lymphocyte count were significantly lower in patients treated with corticosteroids (both *p*<0.001); in contrast, arterial blood pH and serum lactate dehydrogenase were significantly higher in patients receiving corticosteroid treatment (both *p*<0.05). In total, 154 (41.8%) patients with nonsevere COVID-19 received antibiotic therapy; and 58.8% of patients initiated antibiotic treatment no earlier than corticosteroid treatment. The proportion of patients in the corticosteroid group receiving antibiotic therapy was significantly higher than that in the noncorticosteroid group (79.7% vs. 33.9%, *p*<0.001). Quinolones were the most widely used antibiotics in nonsevere COVID-19 cases, followed by cephalosporins, penicillins, macrolides, and carbapenems. All 64 patients who received corticosteroid treatment survived, and 304 cases in the noncorticosteroid group also survived (**Table 2**). Furthermore, hospitalization length and viral shedding time of patients in the corticosteroid group were significantly longer than those in patients in the noncorticosteroid group after adjusting for baseline characteristics, including age, sex, and comorbidities (both *p*<0.05) (**Tables 4** and **5**).

**Table 2:** Comparisons of characteristics between patients treated with and without corticosteroid in nonsevere coronavirus disease 2019 (COVID-19) cases.

Variables	Overall (N=368)	Corticosteroid group (N=64)	Noncorticosteroid group (N=304)	*P*-value
Baseline characteristics				
Age (years)	42.0 (33.0–51.0)	49.5 (39.0–61.0)	41.0 (32.0–50.0)	0.001
Sex, male	180 (48.9)	34 (53.1)	146 (48.0)	0.458
Any comorbidity	79 (21.5)	19 (29.7)	60 (19.7)	0.078
Hypertension	44 (12.0)	12 (18.8)	32 (10.5)	0.065
Diabetes mellitus	27 (7.3)	6 (9.4)	21 (6.9)	0.671
Cardiovascular disease	12 (3.3)	5 (7.8)	7 (2.3)	0.062
Chronic liver disease	8 (2.2)	2 (3.1)	6 (2.0)	0.918
Chronic kidney disease	0 (0)	0 (0)	0 (0)	NA
Cerebrovascular disease	6 (1.6)	5 (7.8)	1 (0.3)	<0.001
Chronic obstructive pulmonary disease	4 (1.1)	2 (3.1)	2 (0.7)	0.141
Malignancy	3 (0.8)	0 (0)	3 (1.0)	1.000
Rheumatic disease	1 (0.3)	0 (0)	1 (0.3)	1.000
Signs and symptoms at admission				
Fever	269 (73.1)	54 (84.4)	215 (70.7)	0.025
Cough	267 (72.6)	50 (78.1)	217 (71.4)	0.272
Expectoration	150 (40.8)	27 (42.2)	123 (40.5)	0.798
Dyspnea	31 (8.4)	6 (9.4)	25 (8.2)	0.763
Temperature, ℃	36.9 (36.5–37.3)	36.9 (36.5–37.6)	36.9 (36.5–37.3)	0.516
Respiratory rate, breaths/min	20.0 (20.0–20.0)	20.0 (20.0–20.3)	20.0 (20.0–20.0)	0.054
Laboratory findings at admission				
Arterial blood pH	7.460 (7.430–7.490)	7.475 (7.445–7.505)	7.460 (7.428–7.487)	0.033
Arterial blood PaO2, mm Hg	92.2 (78.8–115.3)	79.5 (68.1–95.4)	94.3 (82.0–120.0)	<0.001
White blood cell count, *10^9^/L	4.63 (3.58–5.71)	4.59 (3.55–5.95)	4.64 (3.58–5.68)	0.932
Neutrophil count, *10^9^/L	2.84 (2.15–3.69)	3.03 (2.38–3.93)	2.80 (2.13–3.61)	0.082
Lymphocyte count, *10^9^/L	1.17 (0.86–1.60)	0.95 (0.73–1.26)	1.21 (0.89–1.65)	<0.001
Serum lactate dehydrogenase, IU/L	171.8 (142.8–209.0)	182.0 (149.1–242.0)	170.8 (141.9–206.0)	0.047
Chest radiography at admission				
Normal	29 (7.9)	3 (4.7)	26 (8.6)	0.297
Unilateral lesion	85 (23.1)	2 (3.1)	83 (27.3)	<0.001
Bilateral lesion	254 (69.0)	59 (92.2)	195 (64.1)	<0.001
Treatments				
Antiviral therapy	365 (99.2)	64 (100.0)	301 (99.0)	1.000
Interferon	231 (62.8)	36 (56.3)	195 (64.1)	0.235
Lopinavir/Ritonavir	283 (76.9)	47 (73.4)	236 (77.6)	0.469
Arbidol	217 (59.0)	42 (65.6)	175 (57.6)	0.234
Ribavirin	18 (4.9)	5 (7.8)	13 (4.3)	0.383
Chloroquine phosphate	39 (10.6)	9 (14.1)	30 (9.9)	0.322
Antibiotic therapy	154 (41.8)	51 (79.7)	103 (33.9)	<0.001
Quinolones	137 (37.2)	45 (70.3)	92 (30.3)	<0.001
Cephalosporins	10 (2.7)	3 (4.7)	7 (2.3)	0.520
Carbapenems	1 (0.3)	1 (1.6)	0 (0)	0.174
Macrolides	3 (0.8)	1 (1.6)	2 (0.7)	0.437
Penicillins	11 (3.0)	6 (9.4)	5 (1.6)	0.004
Linezolid	0 (0)	0 (0)	0 (0)	NA
Polymyxin	0 (0)	0 (0)	0 (0)	NA
Teicoplanin	0 (0)	0 (0)	0 (0)	NA
Antifungal therapy	1 (0.3)	1 (1.6)	0 (0)	0.174
Voriconazole	1 (0.3)	1 (1.6)	0 (0)	0.174
Noninvasive mechanical ventilation	0 (0)	0 (0)	0 (0)	NA
Invasive mechanical ventilation	0 (0)	0 (0)	0 (0)	NA
ECMO	0 (0)	0 (0)	0 (0)	NA
CRRT	0 (0)	0 (0)	0 (0)	NA

Values are presented as median (IQR) or number (percentage); p values were compared by Chi-square test, Fisher’s exact test, or Mann-Whitney U test.

ECMO, extracorporeal membrane oxygenation; CRRT, continuous renal replacement therapy; NA, not available.

### Comparisons of Characteristics Between Patients Treated With and Without Corticosteroid in Severe COVID-19 Cases

**Table 3** shows comparisons of characteristics between patients treated with and without corticosteroid in severe COVID-19 cases. Among the 82 patients with severe COVID-19, patients treated with corticosteroids were significantly older and were significantly more likely to present comorbidities at admission (both *p*<0.05). Patients with fever were significantly more likely to be treated with corticosteroids (*p*<0.05). The levels of arterial blood pH and serum lactate dehydrogenase in the corticosteroid group were significantly higher than those in the noncorticosteroid group (both *p*<0.05). 71 (86.6%) patients with severe COVID-19 received antibiotic therapy; and 62.7% of patients initiated antibiotic treatment no earlier than corticosteroid treatment. The proportion of patients in the corticosteroid group who received antibiotic therapy was significantly higher than that in the noncorticosteroid group (*p*<0.001). Quinolones were the most widely used antibiotics in severe COVID-19, followed by penicillins, cephalosporins, carbapenems, and linezolid. Of the 62 patients who received corticosteroid treatment, three (4.8%) died; however, 20 patients in the noncorticosteroid group all survived. No significant difference in hospitalization length or viral shedding time was found between the two groups after adjusting for baseline characteristics, including age, sex, and comorbidities (*p*>0.05) (**Table 4** and **5**). The clinical information of fatal cases is shown in **Supplemental Table 2**.

**Table 3:** Comparisons of characteristics between patients treated with and without corticosteroid in severe coronavirus disease 2019 (COVID-19) cases.

Variables	Overall (N=82)	Corticosteroid group (N=62)	Noncorticosteroid group (N=20)	*P*-value
Baseline characteristics				
Age (years)	56.9 ± 15.5	54.5 ± 15.3	64.5 ± 14.1	0.011
Sex, male	48 (58.5)	36 (58.1)	12 (60.0)	0.879
Any comorbidity	49 (59.8)	32 (51.6)	17 (85.0)	0.008
Hypertension	31 (37.8)	21 (33.9)	10 (50.0)	0.196
Diabetes mellitus	18 (22.0)	13 (21.0)	5 (25.0)	0.946
Cardiovascular disease	10 (12.2)	7 (11.3)	3 (15.0)	0.962
Chronic liver disease	3 (3.7)	3 (4.8)	0 (0)	1.000
Chronic kidney disease	1 (1.2)	0 (0)	1 (5.0)	0.244
Cerebrovascular disease	5 (6.1)	2 (3.2)	3 (15.0)	0.169
Chronic obstructive pulmonary disease	6 (7.3)	3 (4.8)	3 (15.0)	0.306
Malignancy	2 (2.4)	2 (3.2)	0 (0)	1.000
Rheumatic disease	1 (1.2)	0 (0)	1 (5.0)	0.244
Signs and symptoms at admission				
Fever	69 (84.1)	56 (90.3)	13 (65.0)	0.019
Cough	66 (80.5)	47 (75.8)	19 (95.0)	0.119
Expectoration	38 (46.3)	29 (46.8)	9 (45.0)	0.890
Dyspnea	27 (32.9)	19 (30.6)	8 (40.0)	0.439
Temperature, ℃	37.3 ± 0.9	37.4 ± 0.9	37.0 ± 0.7	0.069
Respiratory rate, breaths/min	20.0 (20.0-22.0)	20.0 (20.0–22.0)	20.5 (20.0–22.0)	0.943
Laboratory findings at admission				
Arterial blood pH	7.480 (7.442–7.503)	7.483 (7.451–7.509)	7.450 (7.427–7.480)	0.033
Arterial blood PaO2, mm Hg	83.4 (63.3–104.3)	85.8 (64.4–113.3)	71.1 (56.1–83.0)	0.092
White blood cell count, *10^9^/L	5.51 ± 2.74	5.38 ± 2.27	5.90 ± 3.89	0.469
Neutrophil count, *10^9^/L	3.48 (2.48–4.45)	3.53 (2.75–4.59)	2.96 (2.29–3.66)	0.180
Lymphocyte count, *10^9^/L	0.82 (0.63–1.18)	0.80 (0.62–1.14)	0.97 (0.66–1.39)	0.259
Serum lactate dehydrogenase, IU/L	221.4 (176.2–318.8)	247.0 (177.9–349.0)	190.1 (149.0–217.0)	0.017
Chest radiography at admission				
Normal	5 (6.1)	2 (3.2)	3 (15.0)	0.169
Unilateral lesion	18 (22.0)	9 (14.5)	9 (45.0)	0.011
Bilateral lesion	59 (72.0)	51 (82.3)	8 (40.0)	<0.001
Treatments				
Antiviral therapy	82 (100.0)	62 (100.0)	20 (100.0)	NA
Interferon	57 (69.5)	43 (69.4)	14 (70.0)	0.957
Lopinavir/Ritonavir	68 (82.9)	55 (88.7)	13 (65.0)	0.035
Arbidol	47 (57.3)	32 (51.6)	15 (75.0)	0.066
Ribavirin	6 (7.3)	5 (8.1)	1 (5.0)	1.000
Chloroquine phosphate	3 (3.7)	3 (4.8)	0 (0)	1.000
Antibiotic therapy	71 (86.6)	59 (95.2)	12 (60.0)	<0.001
Quinolones	53 (64.6)	47 (75.8)	6 (30.0)	<0.001
Cephalosporins	12 (14.6)	8 (12.9)	4 (20.0)	0.677
Carbapenems	7 (8.5)	7 (11.3)	0 (0)	0.267
Macrolides	1 (1.2)	0 (0)	1 (5.0)	0.244
Penicillins	22 (26.8)	19 (30.6)	3 (15.0)	0.170
Linezolid	6 (7.3)	5 (8.1)	1 (5.0)	1.000
Polymyxin	1 (1.2)	1 (1.6)	0 (0)	1.000
Teicoplanin	1 (1.2)	1 (1.6)	0 (0)	1.000
Antifungal therapy	4 (4.9)	4 (6.5)	0 (0)	0.568
Voriconazole	4 (4.9)	4 (6.5)	0 (0)	0.568
Noninvasive mechanical ventilation	16 (19.5)	16 (25.8)	0 (0)	0.027
Invasive mechanical ventilation	10 (12.2)	9 (14.5)	1 (5.0)	0.461
ECMO	6 (7.3)	6 (9.7)	0 (0)	0.341
CRRT	7 (8.5)	6 (9.7)	1 (5.0)	0.849

Values are presented as median (IQR),mean ± SD or number (percentage); p values were compared by Chi-square test, Fisher’s exact test, independent sample T test, or Mann-Whitney U test.

ECMO, extracorporeal membrane oxygenation; CRRT, continuous renal replacement therapy; NA, not available.

**Table 4:** Comparisons of length of hospitalization in nonsevere and severe coronavirus disease 2019 (COVID-19) cases using propensity score 1:1 matching.

Variables	Before matching				After matching			
	Corticosteroid group	Noncorticosteroid group	*p-*value		Corticosteroid group	Noncorticosteroid group	*p-*value	
Nonsevere group								
Number of cases	64	304			60	60		
Length of hospitalization, days	19.0 (14.0–25.0)	12.0 (9.0–17.0)	<0.001		19.0 (14.0–25.0)	11.5 (9.0–17.0)	<0.001	
Severe group								
Number of cases	59	20			20	20		
Length of hospitalization, days	20.0 (13.0–28.0)	16.0 (11.0–22.5)	0.091		14.0 (11.3–25.5)	16.0 (11.0–22.0)	0.883	

Values are presented as median (IQR) or number; p values were compared by Mann-Whitney U test.

**Table 5:** Comparisons of length of viral shedding in nonsevere and severe coronavirus disease 2019 (COVID-19) cases using propensity score 1:1 matching.

Variables	Before matching			After matching			
	Corticosteroid group	Noncorticosteroid group	*p-*value	Corticosteroid group	Noncorticosteroid group	*p-*value	
Nonsevere group							
Number of cases	63	244		55	55		
Length of viral shedding, days	20.0 (16.0–26.0)	17.0 (13.0–22.0)	0.001	20.0 (16.0–25.0)	17.0 (13.0–22.0)		0.046
Severe group							
Number of cases	53	12		11	11		
Length of viral shedding, days	19.0 (16.0–28.5)	17.5 (13.0–22.8)	0.123	23.0 (16.0–31.0)	18.0 (13.0–23.0)		0.101

Values are presented as median (IQR) or number; p values were compared by Mann-Whitney U test.

## Discussion

To the best of our knowledge, this is the first multicenter retrospective study to describe the use of corticosteroids in treating COVID-19. Our data demonstrate that median daily dose of methylprednisolone in general clinical practice was 56.6 mg and median corticosteroid therapy duration was 5.0 days, which are consistent with recommendations in the updated Chinese management guideline (National Health Commission of the People’s Republic of China). However, indications for corticosteroid treatment in the guideline are flexible, and corticosteroid use, to some extent, relies on physicians’ judgments of patients’ disease progression. Thus, a proportion of nonsevere COVID-19 patients may also receive corticosteroid treatment, although they may not meet the definition of severe disease outlined in the Chinese management guideline. In this study, 64 (17.4%) out of 368 nonsevere cases received corticosteroid treatment, while 62 (75.6%) out of 82 severe cases received corticosteroid treatment. We may speculate that corticosteroids are regarded as one of treatments in the general clinical practice of COVID-19, especially in severe cases.

The validity and rationality of corticosteroids in the treatment of coronavirus diseases have always been controversial. A retrospective study by Chen et al. (2006) found that corticosteroid use was significantly related to lower mortality, longer survival time, and shorter hospital stay in critical SARS patients and was not associated with the incidence of complications. Arabi et al. (2018) found that corticosteroid therapy in patients with MERS was not associated with a difference in mortality after adjustment for time-varying confounders. However, Alfaraj et al. (2019) reported that the use of corticosteroids contributed to increased mortality in MERS patients. Regarding COVID-19, a retrospective study involving 46 patients demonstrated that early, low-dose and short-term application of corticosteroid was associated with a faster improvement of clinical symptoms and absorption of lung focus (Wang et al., 2020); nevertheless, the relatively small sample size and retrospective design limited the interpretation of the study results. Recently, a large-scale and well-designed randomized clinical trial (UK Recovery trial) demonstrated that the proper use of dexamethasone (low-to-moderate dose of 6 mg per day for 10 days) significantly reduced deaths by one-third in patients receiving invasive mechanical ventilation and by one-fifth in other patients who only received oxygen without invasive mechanical ventilation; however, no benefit was observed among those patients who did not require respiratory support (Horby et al., 2020). UK Recovery trial brings a breakthrough in treating severe COVID-19 patients. As far as we know, there has been no attempt of using dexamethasone to treat COVID-19 patients in China; and it is expected that the practical corticosteroid treatment strategy in UK Recovery trial can be verified and popularized worldwide as soon as possible. In this study, the proportion of patients in the severe group receiving corticosteroid treatment was significantly higher than that in the nonsevere group. However, we could not compare our study findings with the UK RECOVERY trial findings since it was not possible to assess the association between corticosteroid use and mortality in our study due to: 1) low death rate among the study cohort; 2) the study was designed and aimed to describe the use of corticosteroids among COVID-19 patients rather than assessing the association between corticosteroid use and mortality outcome; and 3) the type and duration of corticosteroids in this study is different from UK RECOVERY trial.

After the invasion of coronavirus, there are two kinds of inflammatory reaction mechanisms: protective and pathogenic. The pathogenic inflammatory reaction is caused by the massive infiltration of inflammatory cells (monocyte macrophage, neutrophils), release of cytokines, replication of virus, and delay of the interferon reaction, which leads to a decrease in cellular and humoral immunity, significant enhancement of apoptosis, increase in vascular leakage, and the limited virus clearance, and can further result in acute lung injury (ALI), acute respiratory distress syndrome (ARDS), and even death (Channappanavar and Perlman, 2017). Previous cases of SARS and MERS have proven that the cytokine storm caused by the release of a large number of cytokine was the main cause of death (Huang et al., 2005; Kim et al., 2016; Min et al., 2016). Cytokine storm refers to the phenomenon where a variety of cytokines in body fluid, such as TNF-α, IL-1, and IL-6, are produced rapidly after an organism is infected and is an important cause of ARDS and multiple organ failure (Tisoncik et al., 2012). Huang et al. (2005) showed that cytokine storm was involved in the immune damage seen in SARS patients after virus infection. Levels of IFN - γ, IL-18, TGF-β, IL-6, IP-10, MCP-1, MIG and IL-8 were significantly increased in SARS patients; and levels of IL-18, IP-10, MIG and MCP-1 in dead patients were higher than those in living patients (Huang et al., 2005). Two studies by Korean scholars have proven that there is also a significant increase of cytokines (IL-6, IFN-α, IL-8, CXCL10 and CCL5) in patients with MERS, which may lead to a potential cytokine storm (Kim et al., 2016; Min et al., 2016). Huang et al. (2020) reported that the initial plasma concentrations of IL-1 β, IL-7, IL-8, IL-9, IL-10, FGF, GCSF, GMCSF, IFN-γ, IP10, MCP1, MIP1A, MIP1B, PDGF, TNF-α, and VEGF in COVID-19 patients were higher than those in healthy adults, and plasma concentrations of IL-2, IL-7, IL-10, GCSF, IP10, MCP1, MIP1A, and TNF-α in ICU patients were significantly higher when comparing with non-ICU patients. Corticosteroid may have a certain antagonistic effect on cytokine storm. The effect of corticosteroid on systemic cytokine storm has been confirmed in animal models. Saif LJ et al. used pigs infected with coronavirus as models to explore the effect of corticosteroid use on disease models. The results revealed that the number of IFN-γ cytokine secreting cells (CSC cells) in the spleen, tracheobronchial lymph nodes, and blood was lower in the disease model group with dexamethasone intervention than that in the disease model group without corticosteroid intervention, and the secretion of IFN-γ, IFN-α, IL-6, and other cytokines in the disease model group with dexamethasone intervention was inhibited to varying degrees in different periods (Jung et al., 2007; Zhang et al., 2008).

Infections accompanying corticosteroid use in coronavirus diseases are also worthy of attention. Jang et al. (2004) pointed out that six SARS patients who received corticosteroid shock therapy (daily pulsed administration of 0.5–1.0 g of methylprednisolone for two to three days) had multiple hospital infections and suggested careful corticosteroid use with appropriate antibiotic coverage and microbiological monitoring are of great importance in treating SARS patients. Wang et al. (2003) reported the case of a 39-year-old man with fatal fungal infection in multiple organs after repeated, long-term, and high-dose glucocorticoid treatment, who finally died due to ineffective clinical treatment; this case suggests that inappropriate use of large dose and long-term corticosteroid can severely inhibit the immune function of SARS patients. In this study, we observed the proportion of patients receiving antibiotic therapy in the corticosteroid group was significantly higher than in the noncorticosteroid groups, and this was apparent in both severe and nonsevere groups. Moreover, there may be a relationship between corticosteroid and antibiotic use, as 60.9% of patients initiated antibiotic treatment no earlier than corticosteroid treatment. Nevertheless, there is a lack of specific recommendations for antibiotics and antifungal agents in the current guideline, and more studies are warranted to provide evidence regarding this issue.

Furthermore, our results demonstrated a significant prolonged viral shedding time existed in nonsevere patients receiving corticosteroid treatment. A randomized controlled study involving 16 nonsevere SARS patients found that the early application of corticosteroid (within 7 days of onset) was related to the delayed clearance of SARS virus in the plasma; the plasma viral RNA concentration in the hormone intervention group was significantly higher than that in the control group at the 2nd and 3rd weeks (Lee et al., 2004). Another retrospective study on critically ill patients with MERS indicated that after adjusting for confounding factors such as baseline and starting time of corticosteroid therapy, corticosteroid application was significantly associated with delayed clearance time of MERS virus RNA in patients’ plasma; furthermore, there was statistical difference between subgroups with different doses of corticosteroid therapy (Arabi et al., 2018).

Some limitations exist in this study. First, this is a retrospective descriptive study rather than a prospective study; More large-scale randomized placebo-controlled studies should be performed to provide evidence in treating COVID-19. Second, we were unable to directly report the complications of bacterial and fungal infections that were related to corticosteroid use due to the lack of pathogen evidence from blood or sputum culture. Instead, we summarized the proportion of antibiotic and antifungal agents use to indirectly reflect complications of bacterial and fungal infections. Third, because of the limited sample size of fatal COVID-19 cases in this study, we did not use logistic or Cox regression model to explore potential factors related to mortality in COVID-19.

## Conclusion

Our study indicates that corticosteroids are regarded as one of treatments in the general clinical practice of COVID-19. However, corticosteroid use may be accompanied by increased use of antibiotics, longer hospitalization, and prolonged viral shedding.

## Data Availability Statement

Data are available from chenyan99727@csu.edu.cn for reasonable requests.

## Ethics Statement

The studies involving human participants were reviewed and approved by The Second Xiangya Hospital (2020-010). The ethics committee waived the requirement of written informed consent for participation.

## Author Contributions

All authors contributed to data analysis, drafting, and revising the article, gave ﬁnal approval of the version to be published, and agreed to be accountable for all aspects of the work.

## Funding

This project carried out with the support of Hunan Province’s Innovative Novel Coronavirus Pneumonia Emergency Major Project (No. 2020SK3013 and No. 2020SK3014) and Emergency Project of Prevention and Control for COVID-19 of Central South University (No. 502701002).

## Conflict of Interest

The authors declare that the research was conducted in the absence of any commercial or financial relationships that could be construed as a potential conflict of interest.
