# Supplementary material for: Corticosteroid Use in the Treatment of COVID-19: A Multicenter Retrospective Study in Hunan, China
Source: Front Pharmacol. 2020 Aug 12;11:1198. doi: 10.3389/fphar.2020.01198 (PMC7434865; doi:10.3389/fphar.2020.01198)
Supplement: Supplementary file 1 [file Table_1.docx]

**Supplemental Table 1a. Baseline characteristics for matching cohorts when comparing length of hospitalization in non-severe and severe COVID-19 cases**

|  | **Before matching** | | | | **After matching** | | | |
| --- | --- | --- | --- | --- | --- | --- | --- | --- |
| **Variables** | **Corticosteroid group** | **Non-corticosteroid group** | ***p-*value** |  | **Corticosteroid group** | **Non-corticosteroid group** | ***p-*value** |  |
| **Non-severe group** |  |  |  | |  |  |  | |
| **Number of cases** | 64 | 304 |  | | 60 | 60 |  | |
| **Age (years)**  **Sex, male**  **Any comorbidity** | 49.5 (39.0-61.0)  34 (53.1)  19 (29.7) | 41.0 (32.0-50.0)  146 (48.0)  60 (19.7) | 0.001  0.458  0.078 | | 47.9±13.6  31 (51.7)  16 (26.7) | 47.9±14.0  30 (50.0)  18 (30.0) | 0.995  0.855  0.685 | |
| **Severe group** |  |  |  | |  |  |  | |
| **Number of cases** | 59 | 20 |  | | 20 | 20 |  | |
| **Age (years)**  **Sex, male**  **Any comorbidity** | 54.1±15.6  33 (55.9)  30 (50.8) | 64.5±14.1  12 (60.0)  17 (85.0) | 0.010  0.751  0.007 | | 64.2±13.0  12 (60.0)  17 (85.0) | 64.5±14.1  12 (60.0)  17 (85.0) | 0.944  1.000  1.000 | |

**Notes:** Values are presented as median (IQR), mean ± SD or number; p values were compared by Chi-square test, Fisher’s exact test, independent sample T test, or Mann-Whitney U test.

**Supplemental Table 1b. Baseline characteristics for matching cohorts when comparing length of viral shedding in non-severe and severe COVID-19 cases**

|  | **Before matching** | | | | **After matching** | | | |
| --- | --- | --- | --- | --- | --- | --- | --- | --- |
| **Variables** | **Corticosteroid group** | **Non-corticosteroid group** | ***p-*value** |  | **Corticosteroid group** | **Non-corticosteroid group** | ***p-*value** |  |
| **Non-severe group** |  |  |  | |  |  |  | |
| **Number of cases** | 63 | 244 |  | | 55 | 55 |  | |
| **Age (years)**  **Sex, male**  **Any comorbidity** | 50.0 (39.0-61.0)  34 (54.0)  19 (30.2) | 41.0 (33.0-50.0)  106 (43.4)  48 (19.7) | 0.002  0.363  0.072 | | 47.0±13.8  27 (49.1)  14 (25.5) | 47.0±12.8  25 (45.5)  17 (30.9) | 0.989  0.702  0.525 | |
| **Severe group** |  |  |  | |  |  |  | |
| **Number of cases** | 53 | 12 |  | | 11 | 11 |  | |
| **Age (years)**  **Sex, male**  **Any comorbidity** | 54.4±15.7  30 (56.6)  28 (52.8) | 69.1±14.3  6 (50.0)  10 (83.3) | 0.004  0.678  0.107 | | 72.0 (54.0-80.0)  5 (45.5)  10 (90.9) | 74.0(58.0-78.0)  6 (54.5)  9 (81.8) | 0.947  0.670  1.000 | |

**Notes:** Values are presented as median (IQR), mean ± SD or number; p values were compared by Chi-square test, Fisher’s exact test, independent sample T test, or Mann-Whitney U test.
