# Supplementary material for: Corticosteroid Use in the Treatment of COVID-19: A Multicenter Retrospective Study in Hunan, China
Source: Front Pharmacol. 2020 Aug 12;11:1198. doi: 10.3389/fphar.2020.01198 (PMC7434865; doi:10.3389/fphar.2020.01198)
Supplement: Supplementary file 2 [file Table_2.docx]

**Supplemental Table 2. Clinical information of fatal COVID-19 cases in the study**

| **Variables** | **Patient 1** | **Patient 2** | **Patient 3** |
| --- | --- | --- | --- |
| **Baseline characteristics**  **Age (years)** | **62** | **64** | **58** |
| **Sex** | **Male** | **Male** | **Male** |
| **Any comorbidity**  **Hypertension**  **Diabetes mellitus**  **Cardiovascular disease**  **Chronic liver disease**  **Chronic kidney disease**  **Cerebrovascular disease**  **Chronic obstructive pulmonary disease**  **Malignancy**  **Rheumatic disease**  **Symptoms** | **Yes**  **Yes**  **Yes**  **Yes**  **No**  **No**  **No**  **No**  **No**  **No** | **Yes**  **No**  **No**  **Yes**  **No**  **No**  **No**  **Yes**  **No**  **No** | **No**  **No**  **No**  **No**  **No**  **No**  **No**  **No**  **No**  **No** |
| **Fever**  **Cough**  **Expectoration**  **Dyspnea**  **Signs at admission**  **Temperature, ℃**  **Respiratory rate, breaths/min**  **Laboratory findings at admission**  **Arterial blood pH**  **Arterial blood PaO2, mm Hg**  **White blood cell count, *10^9^/L**  **Neutrophil count, *10^9^/L**  **Lymphocyte count, *10^9^/L**  **Serum lactate dehydrogenase, IU/L**  **Chest radiography**  **Treatments**  **Antiviral therapy** | **Yes**  **Yes**  **Yes**  **Yes**  **37.3**  **21.0**  **7.490**  **115.0**  **2.28**  **1.45**  **0.38**  **196.4**  **Unilateral lesion**  **Yes** | **Yes**  **No**  **No**  **No**  **38.7**  **20.0**  **7.502**  **137.1**  **4.58**  **2.79**  **1.42**  **NA**  **Bilateral lesion**  **Yes** | **Yes**  **Yes**  **Yes**  **Yes**  **36.7**  **20.0**  **7.540**  **63.0**  **2.26**  **1.73**  **0.40**  **313.6**  **Unilateral lesion**  **Yes** |
| **Antibiotic therapy**  **Quinolones**  **Cephalosporins**  **Carbapenems**  **Macrolides**  **Penicillins**  **Linezolid**  **Polymyxin**  **Teicoplanin**  **Corticosteroid treatment**  **Type of corticosteroid**  **Route of corticosteroid administration**  **Time from illness onset to corticosteroid therapy, days**  **Time from admission to corticosteroid therapy, days**  **Accumulative dose of corticosteroid therapy, mg**  **Mean daily dose of corticosteroid therapy, mg**  **Duration of corticosteroid therapy, days**  **Antifungal therapy**  **Noninvasive mechanical ventilation**  **Invasive mechanical ventilation**  **ECMO**  **CRRT** | **Yes**  **Yes**  **No**  **No**  **No**  **No**  **No**  **No**  **No**  **Yes**  **Methylprednisolone**  **Intravenously**  **3**  **1**  **120**  **40.0**  **3**  **No**  **No**  **No**  **No**  **No** | **Yes**  **No**  **Yes**  **No**  **No**  **Yes**  **No**  **No**  **No**  **Yes**  **Methylprednisolone**  **Intravenously**  **6**  **5**  **1360**  **97.1**  **14**  **No**  **Yes**  **Yes**  **No**  **Yes** | **Yes**  **No**  **No**  **No**  **No**  **No**  **No**  **No**  **Yes**  **Yes**  **Methylprednisolone**  **Intravenously**  **9**  **2**  **1400**  **77.8**  **18**  **No**  **Yes**  **Yes**  **Yes**  **No** |
| **Death** | **Yes** | **Yes** | **Yes** |

Legends: ECMO - Extracorporeal membrane oxygenation, CRRT - continuous renal replacement therapy, NA – not available.
